# Implementing Problem Management Plus (PM+) in Haiti: qualitative study

**DOI:** 10.1192/bjo.2026.11008

**Published:** 2026-04-07

**Authors:** Michela Marchetti, Ana Carolina Molina, Marcio Gagliato, Orso Muneghina, Pernille Hansen, Faimy Carmelle Loiseau, Giuliana Mazzoni, Corrado Barbui, Marianna Purgato

**Affiliations:** Department of Dynamic and Clinical Psychology and Health Studies, University of Rome La Sapienza, Italy; MHPSS Hub, SOS Children’s Villages Italy, Milan, Italy; The Mental Health and Psychosocial Support Network – MHPSS.net, São Paulo, Brazil; Department of Psychology, Fordham University, New York, USA; Department of Neuroscience, Biomedicine and Movement Sciences, University of Verona, Italy; Independent MHPSS Consultant, La Herradura, Spain; SOS Children’s Villages Haiti, Port-au-Prince, Haiti

**Keywords:** Humanitarian settings, MHPSS, Problem Management Plus (PM+), qualitative research, Haiti

## Abstract

**Background:**

Haiti is experiencing a severe humanitarian crisis characterised by political instability and economic and security hardship. These adversities contribute to significant mental health challenges, which are also exacerbated by poor access to psychological support due to a shortage of specialised professionals. Problem Management Plus (PM+), a scalable and low-intensity intervention developed by the World Health Organization, is based on a task-sharing approach to address the treatment gap by training non-specialist helpers to provide psychosocial support.

**Aims:**

This study aimed to explore the implementation process of PM+ in Haiti, focusing on the barriers and facilitators that influenced its delivery. Specifically, the study focused on understanding the contextual factors affecting intervention accessibility, participant experiences and potential adaptations to enhance its effect.

**Method:**

A qualitative study was conducted across three Haitian cities, where trained helpers delivered PM+. Data were collected through the PSYCHLOPS tool with end-users and via cognitive interviews with stakeholders. Thematic analysis was conducted incorporating Lund’s social determinants of mental health model and Bronfenbrenner’s ecological systems theory to interpret findings.

**Results:**

Sixteen end-users and five stakeholders participated in the study. Key barriers to implementation and its success mainly included economic constraints and safety concerns. Facilitating factors included strong community engagement, adaptive implementation strategies (such as flexible scheduling, remote supervision and culturally responsive adjustments), alongside strong organisational support. End-users described substantial difficulties in managing everyday problems and emotional distress, as reported during pre-intervention qualitative assessments.

**Conclusions:**

PM+ appeared feasible in the Haitian context from an implementation perspective; however, its implementability depends on cultural adaptations, economic considerations and sustained support for facilitators. Addressing systemic barriers and integrating task-sharing interventions within existing health structures could enhance the long-term impact.

Humanitarian settings, characterised by natural disasters, armed conflicts, forced displacement and long-standing economic challenges,^1^ impose severe psychological and social consequences on affected populations.^2^ Humanitarian crises disrupt community structures, displace individuals from their homes and limit access to basic services. The consequences of exposure to humanitarian crises are often long-lasting, leading to heightened levels of mental health symptoms such as depression, anxiety, post-traumatic stress disorder and chronic psychosocial stress.^2–4^ The lack of adequate infrastructure in these settings frequently limits the availability of professional mental health services, creating a significant gap between the need for care and the availability of resources.^5^

Haiti, a country marked by political instability, economic hardship, high insecurity due to armed gangs and frequent natural disasters, is experiencing a severe humanitarian crisis.^6^ Haiti’s current humanitarian emergency is deeply rooted in a complex history of structural inequality, recurrent natural disasters and international economic pressures. The country’s colonial legacy, political interventions and external debt have contributed to long-term fragility and dependence on foreign aid. Decades of political instability and economic exclusion have weakened state institutions and public services, including the health system. Haiti continues to experience escalating humanitarian challenges, including political instability, gang-related violence and economic collapse, resulting in widespread displacement and restricted access to care.^7,8^ These intersecting historical and global forces provide critical context for understanding the chronic humanitarian conditions that shape mental health outcomes in Haiti.

This protracted instability has profound consequences for population mental health. Recent estimates indicate that up to one in three Haitians experiences symptoms of psychological distress, depression or anxiety.^2^ The country has fewer than 2 mental health professionals per 100 000 inhabitants,^2^ and services are heavily concentrated in urban areas. Limited infrastructure, high costs and widespread stigma further restrict access to care, particularly in rural regions.^7,9^

Consequently, mental healthcare is often delivered by non-governmental and international organisations providing short-term humanitarian assistance following emergencies.^7,10^ Cultural and religious beliefs, together with the persistent stigma associated with mental health problems, strongly influence help-seeking behaviour, leading many individuals to rely increasingly on alternative health systems.^11^

Healthcare in Haiti remains critically insufficient, with 40% of the population lacking access to essential services, particularly in rural areas. Half of the country’s health infrastructure is in the capital, leaving many communities underserved.^2,9^ Natural disasters, such as the 2010 earthquake and recurring hurricanes, have further strained healthcare capacity, not only causing physical devastation but also worsening mental health challenges, particularly for those repeatedly exposed to crises.^11^ Without adequate formal care, cultural and religious beliefs heavily influence health-seeking behaviours, leading people to rely increasingly on alternative health systems.^12^ These factors are compounded by widespread poverty, which limits access to psychological support and deepens long-term socioeconomic challenges.^9^ The combination of these adversities sustains a cycle of vulnerability that hinders Haiti’s progress and development.

Mental healthcare, in particular, remains one of the most neglected aspects of Haiti’s healthcare system, creating a significant public health challenge. The country has a critically low number of specialised mental health professionals, with only 2 per 100 000 people, including psychiatrists, mental health nurses, psychologists and social workers.^2^ The sustainability of psychological services represents another significant challenge, because mental healthcare in Haiti is predominantly delivered by non-governmental and international organisations that provide short-term assistance following natural disasters or humanitarian crises.^13^

Given these systemic barriers, there is an urgent need for scalable, sustainable and culturally adapted psychological interventions to address the mental health crisis in Haiti. Developing community-based care models and integrating mental health services into primary healthcare systems would provide pathways to improving access and outcomes for those in need.

Brief psychosocial interventions, such as Problem Management Plus (PM+), can prevent psychological distress related to adversity and support early identification of individuals who may need specialised care. By facilitating referrals for severe cases identified during pre-assessment, PM+ can help reduce the risk of psychological distress escalating into more serious mental health conditions. Through its task-sharing approach, PM+ also addresses the shortage of mental health professionals in Haiti^14,15^ by training non-specialised community members to deliver psychosocial support.^16–18^

PM+ is a psychological, low-intensity, manualised intervention developed by the World Health Organization (WHO) for people aged 16 or above who experience symptoms of depression, anxiety or stress, making it ‘transdiagnostic’ in nature.^19^ The intervention focuses on teaching practical skills for problem-solving, stress management, behavioural activation and social support enhancement, empowering individuals to manage their emotional and practical difficulties effectively. PM+ follows the principle of task-sharing being delivered by trained helpers without a formal background in mental health. Helpers complete training and a period of supervised practice with at least two end-users, and receive constant supervision by specialised mental healthcare staff.^20^ PM+ is designed specifically for delivery in low-resource settings where specialised mental health professionals are scarce.^21^ Its cultural adaptability and cost-effectiveness have made it particularly suitable for use in conflict-affected areas, disaster zones and with underserved and/or vulnerable populations in emergencies.^22–25^ In addition, qualitative evaluations carried out alongside the effectiveness of randomised control trials have demonstrated the acceptability and feasibility of this intervention in a wide range of settings.^26–28^ Furthermore, it can be scalable at country level,^29^ reaching large numbers of individuals and communities across different contexts.^10^ Since its development by WHO, PM+ has been implemented and evaluated in a variety of low- and middle-income countries, including Kenya, Pakistan, Jordan, Turkey and the Central African Republic, and among Ukrainian refugees in Europe. Across these contexts, PM+ has consistently shown feasibility, cultural adaptability and positive mental health outcomes when delivered by trained non-specialists in conflict-affected or resource-limited settings. Despite this growing body of evidence, PM+ had not previously been implemented in Haiti. The Haitian context presents distinctive challenges, including chronic political instability, recurring natural disasters and extreme scarcity of mental health professionals, that differ from the acute crisis environments where PM+ has typically been tested. Implementation of PM+ in Haiti therefore represents the first application of the intervention in a protracted, high-insecurity humanitarian setting in the Caribbean, offering novel insights into its feasibility and cultural adaptation in a Francophone-Kreyòl-speaking, post-colonial context.

This qualitative study examines the implementation of PM+ in Haiti, with particular focus on the barriers and facilitators that shaped its delivery and the contextual factors influencing accessibility and feasibility. Although the effectiveness of PM+ has been established across various low- and middle-income countries, much less is known about its feasibility, adaptability and operational challenges when delivered in complex humanitarian settings. Through analysis of how PM+ was implemented in Haiti through task-shared, community-based delivery, the study seeks to bridge the gap between intervention design and real-world execution, thereby contributing to the growing body of evidence on implementation science in global mental health.

## Method

### Setting

This study was conducted in Haiti, with the PM+ intervention being implemented in Port-au-Prince, Cap-Haïtien and Les Cayes. The study was conducted in community settings across the three cities, where the non-governmental organisation SOS Children’s Villages Haiti (SOS CV Haiti) operates. Due to safety concerns, the implementation took place online or in the SOS CV safe spaces. SOS CV Haiti was responsible for implementation, while the trainers were external experts. Training sessions of PM+ were delivered by 2 trainers to 11 SOS CV Haiti staff members online in English, with simultaneous translation into French. Translation was provided by a bilingual psychologist employed by SOS CV Haiti and trained in PM+. Translation fidelity was monitored throughout the sessions by the lead trainer, who verified the comprehension and equivalence of key intervention terms. The intervention itself was then delivered by helpers in Haiti’s official languages, French and Kreyòl.

The implementation of PM+ in Haiti was led by SOS CV Haiti, in collaboration with SOS CV Italy, which coordinated the research component. Training for PM+ facilitators was conducted online by two WHO-certified trainers external to both SOS CV Haiti and SOS CV Italy, ensuring standardisation and impartiality. The course lasted 5 days (30 h in total) and followed the WHO PM+ manual, covering intervention principles, skill-based exercises, role-play and ethical procedures. Training was delivered in English with simultaneous translation into French, followed by a supervised practice phase in which each helper was asked to conduct two full PM+ cases under the trainers’ remote supervision. Ongoing supervision was maintained weekly throughout the implementation phase via secure online platforms, focusing on case review, problem-solving and emotional support for facilitators.

The implementation of PM+ in Haiti took place between October 2023 and October 2024. Qualitative data collection was conducted from June to November 2024, but data analysis started only in March 2025 due to coordination challenges associated with the humanitarian crisis.

The project was supported by Elrha’s Humanitarian Innovation Fund (grant no. 81275), which covered training, supervision and logistical costs but did not include financial incentives for helpers, end-users or stakeholders. Participation was entirely voluntary. End-users were recruited through existing SOS CV community programmes, with outreach conducted by local helpers familiar with the environment and the families they serve. Stakeholders were invited among PM+ implementers and trainers to provide reflective insights on the programme’s delivery and adaptation in the Haitian context.

The protocol for this study has been registered in Open Science Framework (https://doi.org/10.17605/OSF.IO/SH7VT).

The authors assert that all procedures contributing to this work comply with the ethical standards of the relevant national and institutional committees on human experimentation, and with the Helsinki Declaration of 1975 as revised in 2013. Ethical approval was obtained from the Technical-Scientific Committee of SOS CV Italy. In Haiti, the protocol was reviewed and authorised by SOS CV Haiti’s national office, which provided local administrative and ethical clearance in line with institutional policies.

### Local adaptation

Given the country’s limited exposure to scalable psychosocial interventions and the strong stigma surrounding mental health, the implementation of PM+ in Haiti required local adaptation to address both systemic and cultural challenges. In Haiti, psychological services are often associated with private clinics, which many cannot afford, or under-resourced public centres, many of which are no longer operational.^30^ Additionally, widespread insecurity has led to a shortage of trained professionals, necessitating a shift in how care is delivered.^31^

To respond to these realities, the team adapted the intervention by emphasising task-shifting to trained community members and the delivery of care outside of traditional clinical settings. PM+ sessions were conducted in community-based and semi-formal environments, including safe spaces provided by SOS CV Haiti, participants’ homes when feasible and, during periods of insecurity, through remote or telephone sessions. This flexible approach ensured accessibility and continuity of care in a fragile humanitarian context.

PM+ materials were translated into French and Haitian Kreyòl to ensure accessibility for both helpers and end-users. Beyond translation, no formal modification of the manual was undertaken. Helpers drew on their familiarity with local customs and everyday language to explain key intervention concepts in culturally meaningful ways during delivery. These informal, delivery-level adjustments allowed for contextual relevance while maintaining fidelity to the PM+ model.

Recognising that Haitians are not typically familiar with *ad hoc* psychosocial support, the team in Haiti also conducted extensive community engagement and education efforts to introduce the PM+ model in a way that resonated with local values and needs. Community engagement and education were carried out through the existing SOS CV Haiti network of family-strengthening and child-protection programmes. Local helpers (staff members already working within these programmes and familiar with community members) introduced the PM+ model during regular meetings. These interactions focused on explaining the purpose of the intervention, its voluntary nature and the types of everyday stressors that it addressed. Programme coordinators also held preparatory discussions with community representatives and caregivers to ensure cultural appropriateness and transparency. These relational engagement processes, rather than public campaigns, were essential in fostering community trust and facilitating participant recruitment.

These adaptations were crucial in building trust and acceptance. During debriefing interviews, the majority of end-users described the sessions as useful, comforting or motivating. This proportion was derived from qualitative frequency counts rather than a formal quantitative measure, and should be interpreted as indicative of acceptability rather than statistical satisfaction. This experience underscores how culturally sensitive and context-specific adaptations are essential for the successful implementation of psychosocial interventions in low-resource and high-stigma environments.

### Participants

As end-users of the PM+ intervention, we included adult participants (over 18 years old) based in Haiti who were proficient in French and/or Kreyòl.

Additionally, we included stakeholders who provided insights into the barriers, facilitators and overall progress of the project’s implementation. The stakeholder group comprised two helpers from SOS CV (who worked with end-users exposed to the humanitarian emergency and were proficient in French and English), two PM+ trainers and one mental health and psychosocial support (MHPSS) expert who provided training on the supervision of PM+. Prior to data collection, SOS CV engaged community leaders and family representatives to introduce the project, strengthen community trust and support participant recruitment. This engagement facilitated local acceptance of the intervention and contributed to its successful implementation.

Qualitative measures from the end-users were collected by helpers trained in PM+ via the PSYCHLOPS tool.^32^ Qualitative data from the stakeholders were collected by the first author, who conducted qualitative interviews that followed a script and lasted 30 min each.

Before the implementation of PM+, end-users received an explanation of the intervention and were requested to sign both an informed consent form for participation and a consent form for processing of personal data. Verbal consent was witnessed and formally recorded.

### Qualitative tools and interviews

Qualitative data were collected through cognitive interviews^33^ and the PSYCHLOPS tool,^32^ a validated measure designed to capture self-identified problems and their impact on participants’ lives. This tool includes open-ended questions that explore individuals’ adversities, their severity and their effect on daily functioning and emotional well-being.

PSYCHLOPS was administered once, during the pre-assessment phase of PM+, as part of the methodology, to document participants’ self-perceived problems, stressors and functioning difficulties prior to receiving any intervention. The tool was used qualitatively to elicit personal accounts of psychological and practical adversities rather than to assess change over time, due to its use only at baseline. These data provided a foundation for understanding the main psychosocial needs and everyday challenges faced by end-users in the Haitian context. This structure may have shaped the data by emphasising participants’ prioritised psychosocial problems and functional impairments.

End-user PSYCHLOPS data offered a bottom-up perspective on lived psychosocial challenges, contextual barriers and priorities for care, with stakeholder interviews contributing a top-down perspective on organisational and systemic enablers or constraints to implementation. The integration of these two perspectives allowed us to interpret findings across ecological levels (individual, community, systemic) and provided a comprehensive understanding of how individual experiences of distress intersect with structural and cultural conditions shaping access to mental healthcare in Haiti.

Insights from stakeholders were obtained through five cognitive interviews guided by a script developed to explore the implementation of PM+ within the Haitian context (see Supplementary Material available at https://doi.org/10.1192/bjo.2026.11008). Participants were asked to discuss barriers, facilitators and the overall progress of the project’s implementation. Specific questions explored challenges and barriers to accessing psychological interventions in Haiti, considering cultural, economic, geographical and social factors. Participants were also asked to share the obstacles they encountered within this context. Additionally, they were asked to identify factors or resources that have been, or could potentially help, improve access to, psychological interventions. This included discussions on support systems, local resources, cultural approaches and community-driven solutions that have contributed to the successful implementation of psychological interventions. All interviews were conducted in Haitian Kreyòl or French, transcribed verbatim and translated into English by bilingual Haitian research assistants familiar with PM+ terminology and local idioms. Translations were reviewed collaboratively by bilingual team members to ensure accuracy and preserve semantic nuance. Any ambiguous terms were discussed until consensus was reached among the coding team.

Data from end-users was collected through a survey administered via Qualtrics XM Platform 2017 (a web-based platform used on Windows and macOS; Qualtrics, Provo, Utah, USA; https://www.qualtrics.com), whereas interviews with stakeholders were conducted via Microsoft Teams 2017 (used on Windows and macOS; Microsoft Corporation, Redmond, Washington, USA; https://teams.microsoft.com). All data were securely stored in a protected database accessible only to the research team.

### Analysis

Data collection and analysis were based on multiple credibility strategies to ensure methodological safety. We employed the following strategies: a thematic analysis approach was used to analyse qualitative data from PSYCHLOPS and cognitive interviewing of the key informants. Responses were reviewed and coded iteratively to identify recurring themes and patterns. The analysis followed Braun and Clarke’s six-phase framework, beginning with familiarisation through repeated reading of transcripts, generating initial codes, searching for themes, reviewing themes, defining and naming themes and writing up.^34^ These codes were then grouped into broader themes to capture core patterns across the data. Reflexivity was maintained throughout the process, with attention given to researchers’ positionality and potential biases. Two independent researchers performed coding manually to ensure reliability and minimise bias, with discrepancies resolved through discussion until consensus was achieved. The research team included both Haitian and European members, as well as individuals affiliated with SOS CV. Reflexive discussions were held throughout the analysis to consider how cultural background and organisational proximity might influence coding and interpretation, and themes were reviewed collaboratively to reduce potential bias. Data saturation was reached when no new themes emerged, ensuring the comprehensiveness of the analysis. Saturation was assessed pragmatically by monitoring whether additional interviews had generated new thematic insights. Within this sample, no new major themes emerged; however, theoretical saturation was not claimed and the findings reflect themes identified within this specific group.

Thematic analysis identified seven overarching themes: (a) economic hardship, (b) health concerns, (c) family responsibilities, (d) emotional distress, (e) safety concerns, (f) cultural mismatches and (g) systemic barriers. Each theme comprised subcategories including unemployment, food insecurity, caregiving burden and insecurity, which reflect the multi-level determinants of psychological well-being in Haiti.

**Fig. 1:**
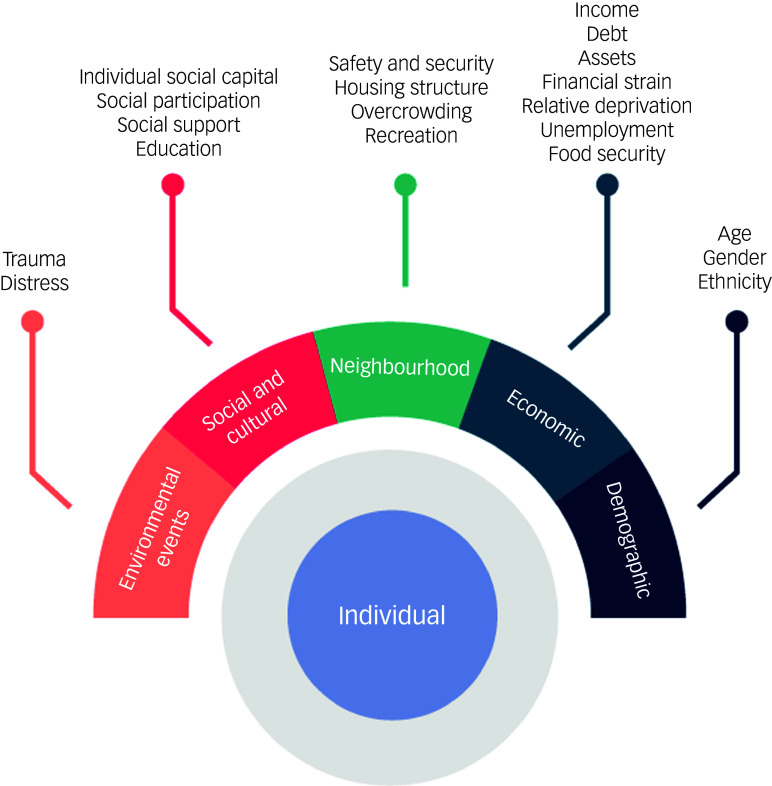
Lund’s model of social and cultural determinants of mental disorders (adapted from Lund et al^35^).

In addition, interpretation of the results was grounded in a theoretical framework that integrated Lund’s theory on the social determinants of mental health^35^ with Bronfenbrenner’s ecological systems theory.^36^ Lund’s theory, which examines mental health through the lens of social determinants, emphasises the impact of social factors on psychological well-being. Factors are organised into five domains: environmental events, social and cultural, neighbourhood, economic and demographic (see Fig. 1).

**Fig. 2:**
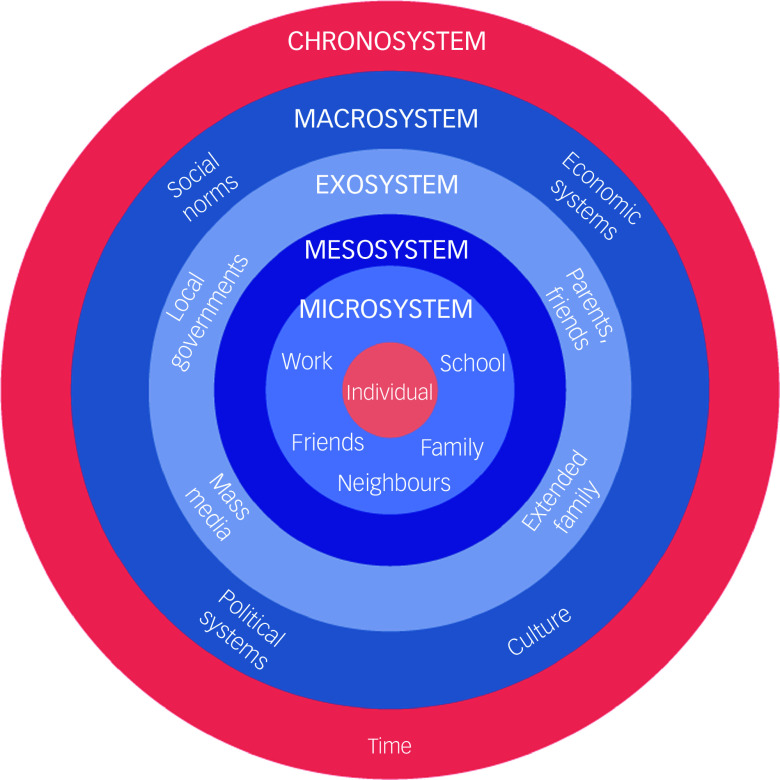
Bronfenbrenner’s ecological systems theory.^36^

Bronfenbrenner’s ecological systems theory provides a parallel perspective by placing human development in nested environmental systems, ranging from the microsystem (e.g. family and peers) to the macrosystem (e.g. cultural norms and policies; see Fig. 2).

Together, these models allow a more detailed explanation of the findings, locating individual experiences within social environments and systemic conditions.

## Results

### Qualitative results from end-users: anchoring in Lund’s and Bronfenbrenner’s theoretical frameworks

PSYCHLOPS was administered to identify adversities by the helpers to 16 end-users (15 females, mean age 37 years) who completed the pre-assessment of the PM+ protocol. Qualitative data analysis from end-users identified seven interconnected themes that summarise the multidimensional adversities of individuals in Haiti (see Table 1 for a comprehensive overview).

**Table 1:** Themes of qualitative analysis within Lund’s framework and Bronfenbrenner’s model

Theme	Findings from end-users and stakeholders	Lund’s framework	Bronfenbrenner’s model
Economic hardship	Unemployment, financial struggles and inability to meet basic needs. Stakeholders noted that indirect costs (e.g. transportation) limited participation in Problem Management Plus (PM+).	Economic domain: poverty directly contributes to mental distress by increasing stress, anxiety and hopelessness.	Exosystem and macrosystem: economic policies, job availability and financial barriers impact individuals indirectly.
Health concerns	Physical and mental health problems (both personal and family-related) intensified emotional distress. Limited access to healthcare.	Neighbourhood domain: poor health reduces work capacity, increasing poverty and further damaging mental health.	Microsystem and exosystem: Individual health conditions interact with limited healthcare access at the community level.
Family responsibilities	End-users struggled with caregiving and educational responsibilities. Stakeholders noted that PM+ required extra efforts to adapt to family constraints.	Economic domain: financial instability limits the ability to provide for children, increasing emotional burden.	Microsystem and mesosystem: family relationships, childcare and education access affect well-being.
Daily functioning	Fatigue, sleep problems and difficulty in maintaining routines were common among end-users.	Environmental events domain: mental distress reduces productivity and functional capacity, worsening economic hardship.	Microsystem: individual psychological challenges affect everyday behaviours.
Emotional distress	Feelings of sadness, helplessness and being overwhelmed due to socioeconomic struggles.	Economic domain: poverty-related stressors increase the risk of depression and emotional suffering.	Microsystem and mesosystem: family and community stressors influence emotional well-being.
Social environment	End-users reported family tensions and uncomfortable living conditions. Stakeholders highlighted social distrust due to insecurity.	Social and cultural domain: unstable social relationships worsen mental health outcomes.	Microsystem and exosystem: housing, family dynamics and community relationships shape stress levels.
Compound stressors	Multiple stressors (e.g. unemployment, health issues, family conflicts) intensified distress. Stakeholders noted systemic gaps (e.g. lack of referral networks, political instability).	Social and cultural domain: the accumulation of stressors makes it harder to break the cycle of poverty and mental distress.	Chronosystem and macrosystem: long-term instability (economic, political, social) affects mental health resilience.
Safety concerns	Political instability, crime and mobility restrictions made PM+ implementation difficult. End-users also faced insecurity at home.	Social and cultural domain: fear and instability contribute to chronic stress, worsening mental health outcomes.	Exosystem and macrosystem: community and national security issues limit access to mental health services.
Cultural mismatch	Stakeholders reported that PM+’s non-directive approach conflicted with Haitian norms of advice-giving.	Social and cultural domain: psychological interventions must align with cultural expectations to be effective.	Macrosystem: cultural norms and societal expectations shape how mental health support is received.
Systemic barriers	Weak referral systems and connectivity issues limited programme effectiveness.	Environmental events domain: structural weaknesses prevent people from accessing mental health services.	Exosystem and macrosystem: institutional gaps and infrastructure challenges impact intervention success.
Facilitators	Despite barriers, facilitators adapted flexibly, community members were receptive and organisations provided support (e.g. virtual tools).	Economic domain: economic and social support systems help mitigate the negative effects of poverty on mental health.	Mesosystem and macrosystem: support networks, programme flexibility and community engagement enhance mental health resilience.

Economic hardship and family responsibilities arose as interconnected challenges, with all participants citing unemployment and poverty as major stressors. Most of them faced significant challenges in meeting their family’s needs, experiencing a sense of powerlessness due to lack of employment. At the same time, financial constraints limited their ability to provide essential resources (e.g. education, healthcare and daily necessities). As one participant noted, ‘Chômage, difficulté à répondre aux besoins de ma famille’ (Unemployment, difficulty meeting family needs), while another highlighted the cumulative impact of livelihood loss, stating ‘Chômage, perte des activités de commerce’ (Unemployment, loss of business activities) and a third participant declared, ‘Situation économique difficile – trop de responsabilités’ (Difficult economic situation – too many responsibilities). Lund’s theory links macroeconomic factors such as unemployment to psychological distress, whereas Bronfenbrenner’s framework situates these issues within broader economic systems. Together, these models illustrate how economic instability and family stress shape mental health outcomes, affecting caregiving roles.

Health-related problems were identified as another major theme, either physically or mentally, exacerbating existing adversities. Such health-related problems also affect other family members, increasing the general stress level. One notable example involves a mother who stated, ‘La maladie de sa fille: ne pas dormir les soirs’ (The illness of her daughter that doesn’t let her sleep at night), whereas others reported ongoing physical strain such as ‘Maux de tête et insomnie fréquents’ (Frequent headaches and insomnia). Lund’s model can frame this sentence, explaining how poor health conditions reinforce poverty and exacerbate mental health challenges. Bronfenbrenner’s model frames this phenomenon in the microsystem and exosystem, where personal health concerns overlap with larger issues of the availability of healthcare.

Most participants reported a significant impairment of daily functioning, making even routine activities such as waking up in the morning or following planned activities difficult. Fatigue and psychological distress, often linked to sleep disturbances, were the most cited difficulties. For example, one participant stated, ‘Me lever le matin, le problème de sommeil’ (Waking up in the morning, sleep problems), highlighting the widespread difficulty in maintaining daily activities. Others described challenges in fulfilling everyday caregiving tasks, including ‘Ne pas pouvoir amener sa fille à l’école à l’heure’ (Not being able to take her daughter to school on time) and ‘Prendre soin des enfants en les accompagnant dans leurs activités’ (Taking care of children and accompanying them in daily activities). Emotional distress was another recurring theme, with participants frequently expressing feelings of sadness and being overwhelmed, as reflected in phrases such as ‘très mauvais état émotionnel’ (very bad emotional state). These emotional challenges often stemmed from the interplay among family, socioeconomic and security factors, predicting that mental distress reduces productivity, as described by Lund. Bronfenbrenner’s micro- and mesosystem illustrate how psychological challenges and social stressors shape daily behaviour and emotional well-being.

Finally, compounding stressors were a recurring theme in most answers. Participants often described how different issues, such as loss of livelihood activities and unemployment, cumulatively increased their overall psychological status. For instance, one participant stated ‘Chômage, perte des activités de commerce’ (Unemployment, loss of business activities), noting the cumulative nature of these threats. Furthermore, another emphasised the combined impact of joblessness and caregiving responsibilities, noting ‘pas de travail, impossibilité à prendre soin de mes enfants’ (no work, inability to take care of my children). Lund’s model shows how interrelated stressors block efforts to alleviate poverty and psychological distress. Bronfenbrenner’s macro- and chronosystem emphasise the impact of prolonged economic, political and social uncertainty on mental health resilience.

### Stakeholder qualitative results: anchoring in Lund’s and Bronfenbrenner’s theoretical frameworks

Five stakeholders were interviewed to assess the implementation process of PM+ in Haiti. Although programme implementation encountered challenges related to Haiti’s social, economic and systemic barriers, qualitative interviews provided valuable insights to inform further adaptation and improvement of its implementation in similar contexts (see Table 1). Table 1 maps emergent themes onto the levels of Lund’s and Bronfenbrenner’s frameworks to support interpretation, without suggesting causal or empirical linkages.

Safety issues arose as a persistent challenge, severely impacting end-users’ and helpers’ ability to access the programme. Sociopolitical instability limited mobility, hindered communication and promoted distrust, thus presenting strong barriers to programme continuity. As one helper described, ‘Les plus grands obstacles sont les problèmes de l’insécurité, les problèmes économiques marqués par la pauvreté et le chômage’ (The greatest obstacles are problems of insecurity and economic hardship marked by poverty and unemployment). An MHPSS expert similarly noted, ‘The biggest barrier for everyone… is safety concerns. It affects communication, mobility, and trust in other people’. Lund’s theoretical framework highlights that anxiety, and instability, may exacerbate chronic stress, leading to deteriorating mental health status. Further, Bronfenbrenner’s exo- and macrosystem describe how security concerns at the community and national levels influence access to mental healthcare.

Haiti’s economic vulnerability further exacerbated the challenges related to accessing PM+. Although PM+ was offered free from any direct financial cost, indirect costs such as transport to reach the implementation setting posed some challenges. For the individuals responsible for managing basic subsistence needs, PM+ sessions were not considered a top priority. As one helper explained, ‘Souvan, moun yo pa ka vin paske yo pa gen lajan pou transpò’ (Often, people cannot come because they have no money for transport). This aligns with Lund’s conceptual framework illustrating how restricted finances hinder participation in psychological interventions. Moreover, Bronfenbrenner’s exo- and macrosystem illustrate how financial policy and infrastructure influence access to various interventions.

Another challenge, primarily cultural, was the PM+ emphasis on problem-solving rather than providing advice. This could conflict with Haitian cultural norms, where advice-giving is a fundamental aspect of interpersonal support.^37^ As one trainer reflected, ‘Sometimes the helpers were confused because they felt they had to advise participants, but PM+ asks them to let people find their own solutions’. This misalignment necessitated additional efforts for facilitators to foster trust and adapt the programme’s implementation. Lund’s model underscores the importance of aligning psychological interventions with cultural expectations to ensure their effectiveness, whereas Bronfenbrenner’s macrosystem framework highlights the influence of societal norms on the provision and reception of mental health support.

Despite the numerous barriers, several facilitating factors contributed to the successful implementation of PM+ in Haiti. These included the flexibility and resilience exhibited by facilitators, the community’s receptiveness, institutional support and the inherent adaptability within the programme’s implementation process. As one helper highlighted, ‘Les clients se sentent plus dans le bain d’être proche de professionnels de la santé mentale sans rien payer’ (Clients feel more involved, as if they were close to mental health professionals without having to pay). Supervisory commitment was also crucial to the programme’s success. Although facilitators faced challenges such as connectivity issues and security concerns, their determination to overcome these obstacles ensured the programme’s sustainability. One trainer described this adaptability vividly: ‘We had to fix the plane while it was flying’. Participants provided largely positive feedback on the PM+ intervention, reflecting its ability to engage and support them effectively.

Support from the trainers, such as providing online training and flexibility in scheduling, played an essential role in addressing logistical challenges and maintaining programme continuity. Facilitators and trainers demonstrated significant adaptability, employing creative solutions to handle issues such as interrupted schedules and inconsistent attendance, which was crucial for sustaining engagement. Lund’s model highlights the importance of economic and social support structures in mitigating the adverse effects of poverty on mental health. At the same time, Bronfenbrenner’s meso- and macrosystem frameworks underscore the role of support networks, programme flexibility and community involvement in strengthening mental health resilience.

## Discussion

This qualitative study explored the implementation of PM+ in the latest escalation of an ongoing humanitarian crisis in Haiti. The introduction of PM+ in Haiti addressed the urgent need for scalable and accessible MHPSS interventions in contexts where sociopolitical instability, violence and recurrent natural disasters are commonplace. Given Haiti’s significant shortage of mental health professionals, the programme followed the task-shifting approach to train non-specialist PM+ helpers, expanding service provision. Despite challenges, the project has successfully pioneered the first fully online delivery of PM+ training, demonstrating its feasibility in high-severity settings where in-person interventions may not be possible. The successful completion of all PM+ training and supervision online, amid widespread insecurity, demonstrates the feasibility of remote capacity-building for psychosocial interventions in high-risk contexts. One facilitator reflected, ‘Even when we couldn’t meet, supervision online helped us stay connected and motivated’. This highlights the critical role of sustained supervisory commitment in maintaining programme quality and morale.

The findings underscore the fact that successful implementation in humanitarian settings depends not only on the clinical soundness of interventions but also on their feasibility, adaptability and responsiveness to context. This study demonstrates how PM+ can be effectively delivered by non-specialists through flexible, community-based mechanisms even amid sociopolitical instability. By focusing on implementation processes, the Haitian experience provides actionable insights for scaling evidence-based psychosocial interventions, offering practical guidance for clinicians, programme implementers and policy-makers working in crisis-affected environments.

The demographic profile of participants – 15 out of 16 end-users were female, with a mean age of 37 years – reflects broader patterns in help-seeking behaviours observed in humanitarian and community-based psychosocial programmes, where women are often more likely than men to engage with mental health services and community interventions. In the Haitian context, women frequently hold primary caregiving roles and are more accessible to community helpers during recruitment activities. Although this gender imbalance is common in psychosocial research, it may limit the generalisability of findings to male populations, whose experiences of distress and coping might differ. Future PM+ adaptations in Haiti could explore targeted outreach strategies to increase men’s participation and ensure more gender-balanced representation.

The project’s success is further contextualised through qualitative findings that highlight the cyclical relationship between economic hardship and mental health, which is in line with Lund’s theory, emphasising a cyclical relationship between economic hardship and psychological distress. His cycle is particularly evident in the context of Haiti, where systemic poverty, sociopolitical instability and limited access to healthcare compound these challenges. The implications of this dynamic reinforce the urgent need for interventions that simultaneously address the social determinants of mental health and psychological well-being.^9,38,39^ For example, several studies have investigated how targeted interventions addressing economic hardship can have significant mental health benefits. In their study, Zimmerman and colleagues^40^ investigated how cash transfer programmes alleviate financial strain and positively affect young adults’ mental health. Their findings indicate that financial support mechanisms have the potential to break the cycle of poverty and psychological distress by enhancing economic security and mitigating the stressors associated with material deprivation. In addition, Lund and colleagues^41^ explored solutions to poverty reduction with self-regulation interventions to alleviate adolescent depression and anxiety in different countries. The programme outlined in the research is a significant illustration of the potential of large-scale interventions to effectively serve the long-running cycle connecting economic disadvantages and poor mental health.

Bronfenbrenner’s ecological systems theory^36^ further contextualises these experiences by highlighting how environmental factors at multiple levels interact to shape mental health outcomes. At the microsystem level, individual distress, characterised by symptoms such as sleep disturbances, fatigue and emotional exhaustion, directly influences daily functioning.^42^ Family instability and caregiving responsibilities heightened stress at the mesosystem level, underscoring the interdependency of economic security and mental well-being. The exosystem demonstrated how systemic barriers, such as transportation costs and limited healthcare access, indirectly constrained mental health service utilisation, further emphasising the structural dimensions of psychological distress.^11,43^ The macrosystem level presented significant obstacles to intervention success, particularly within the Haitian sociopolitical landscape, with economic fragility and safety concerns that restricted mobility and communication. These findings align with previous studies on mental health service delivery in conflict-affected regions, where infrastructural deficits and security risks presented significant obstacles.^31,44^ The chronosystem further contextualises these findings by illustrating how ongoing economic and political challenges hinder long-term intervention sustainability, posing significant risks to mental health service continuity.

Integrating Lund’s and Bronfenbrenner’s frameworks provided a multidimensional lens for understanding psychosocial distress and intervention processes in crisis settings. Whereas Lund’s model foregrounds the structural and economic determinants of mental health inequities, Bronfenbrenner’s ecological systems theory focuses on how individuals interact with their immediate, community and societal environments. In the context of humanitarian MHPSS, these frameworks are mutually reinforcing: together, they illuminate how macro-level instability and resource deprivation (Lund) cascade into meso- and micro-level disruptions in caregiving, social support and emotional regulation (Bronfenbrenner). This integration avoids both structural reductionism and individual essentialism, offering a holistic perspective on how social determinants and ecological interactions jointly shape the experience of distress and recovery.

Despite these barriers, key enabling factors contributed to the success of PM+. The resilience and flexibility of facilitators were crucial in mitigating operational and safety challenges. Their problem-solving skills, commitment and capacity to navigate logistical constraints ensured sustained engagement, even during systemic instability. The observed resilience and flexibility among facilitators were largely fostered by the structured training and consistent supervision provided throughout the programme. The combination of standardised instruction, practical case rehearsal and continuous supervisory contact strengthened their confidence in adapting PM+ delivery to local challenges, including safety concerns and logistical constraints. This indicates that facilitator adaptability in humanitarian contexts is not merely an intrinsic quality but can be effectively cultivated through structured mentorship and competency-based support.^42–44^

Additionally, organisational support, such as virtual tools and flexible scheduling, facilitated intervention accessibility, demonstrating the importance of flexible implementation approaches in humanitarian contexts. These findings emphasise the necessity of integrating economic support mechanisms within psychological interventions to break the poverty–mental health cycle. Addressing financial constraints could significantly improve intervention uptake and long-term outcomes.^42^

This study acknowledges several limitations. The use of qualitative data, while offering a detailed understanding of participants’ lived experience, restricts the generalisability of results. The gender imbalance among end-users, with 15 of 16 participants being women, reflects the current literature in the field.^24,45^ However, this should be carefully considered in the interpretation of results, because it may further limit the transferability of the findings. Men’s experiences of psychosocial distress, help-seeking and coping may differ and were under-represented in this sample. In addition, the stakeholder sample consisted primarily of individuals directly involved in PM+ implementation, including helpers, trainers and an MHPSS expert affiliated with the implementing organisation. The absence of external stakeholders, such as Ministry of Health representatives, independent clinicians or community leaders not employed by SOS CV, may have constrained the range of perspectives captured and increased the risk of organisational or implementation-related bias. Technical issues, such as connectivity problems and inconsistency in session presentation, may have impacted the programme’s effect. Moreover, the study was conducted during a severe flare-up within a long-term humanitarian emergency, leading to the inclusion of fewer participants. A further limitation concerns the fact that not all implementation outcomes, as defined by Proctor et al^46^, were systematically collected and reported using standardised quantitative indicators (e.g. number approached, uptake, session completion, drop-out and fidelity). Within the constraints of this project and the ongoing humanitarian emergency, we report all implementation-related information that was feasibly available, primarily through qualitative accounts from field experience and stakeholder perspectives. Although a limitation, this also serves as a strength because it highlights the feasibility of implementing PM+ in real-time emergency settings, providing valuable insights regarding implementing scalable interventions under challenging circumstances. Future research should include quantitative evaluations and longitudinal follow-ups to assess the sustained impact of PM+ in Haiti. The resurgence of violence and mobility restrictions during data collection constrained both sample size and interview consistency, occasionally affecting the depth and audio quality of some recordings. Moreover, the study design did not include longitudinal follow-up, limiting our ability to explore how the perceived benefits of PM+ evolved over time. Future work should incorporate follow-up assessments and consider mixed-methods approaches to examine the sustainability of psychosocial gains. Building on the feasibility demonstrated here, hybrid models combining remote and in-person PM+ delivery may offer a practical path forward in contexts of recurrent instability. Online supervision and tele-delivery could help maintain programme continuity when face-to-face sessions are disrupted. Finally, the structured, problem-solving and empathy-based framework of PM+ appeared to mitigate aspects of mental health stigma by normalising emotional distress as a response to adversity, and by framing support within everyday problem management. This feature aligns with local values of mutual aid, and could inform broader community-level stigma-reduction strategies in Haiti.

By integrating the findings, this project contributes to the growing body of humanitarian research and innovation demonstrating that scalable, evidence-based mental health interventions can be adapted to complex emergency settings. The feasibility of PM+ delivery in Haiti not only enhances access but also creates a model for its use in other crisis-affected regions where logistic and security concerns often constrain face-to-face interventions. These qualitative results may also inform future quantitative analyses, including preliminary reflections on cost and economic sustainability, thereby supporting more comprehensive planning and evaluation of such interventions.

## Supporting information

Marchetti et al. supplementary material Marchetti et al. supplementary material

## Data Availability

The data that support the findings of this study are available from the corresponding author, M.M., upon reasonable request.

## Supplementary material

The supplementary material is available online at https://doi.org/10.1192/bjo.2026.11008
