# Supplementary material for: Implementing Problem Management Plus (PM+) in Haiti: qualitative study
Source: BJPsych Open. 2026 Apr 7;12(3):e101. doi: 10.1192/bjo.2026.11008 (PMC13107291; doi:10.1192/bjo.2026.11008)
Supplement: Marchetti et al. supplementary material [file S2056472426110084sup001.docx]

**Supplementary materials**

1. Introduction

*Thank you for taking the time to speak with us today. We’re interested in understanding your experience with the implementation of PM+, what worked well, what challenges you faced, and what could be improved. There are no right or wrong answers; we just want your honest perspective. This interview will take about 20–30 minutes.*

2. Context

Q1. Can you briefly describe your role and involvement in the implementation of PM+?

*ex:* What were your main responsibilities? How long have you been involved?

3. Facilitators (What worked well)

Q2. From your experience, what aspects of the PM+ implementation went particularly well?

*ex:* What factors helped things run smoothly? Were there any supports (training, leadership, resources, community engagement) that made it easier?

4. Barriers (What did not work well)

Q3. What were the main challenges or barriers you encountered during the implementation?

*ex:* What made it difficult to deliver or sustain PM+? Were there issues with staff capacity, supervision, community buy-in, or system-level factors?

5. Adaptations

Q4. How did you or your team address these challenges?

*ex:* Did you make any adaptations to the PM+ approach? What changes or solutions were most helpful?

6. Reflection

Q6. Looking back, what advice would you give to others planning to implement PM+ in a similar context?

*ex:* What would you do differently next time?

8. Closing

*Thank you again for sharing your insights. Your feedback is very valuable in helping us understand how to improve PM+ implementation.*
